# 

**Published:** 1894-01-20

**Authors:** 


					Tbk hospital. {J January 20th, 1894.
WITH EXTRA NURSING SUPPLEMENT.
Per Annum, 8s. 6d.t post free.
Monthly Farts, 6d.; post free, 8cL
Ditto per Annum, 8s., post free.
jlascqw Western Infi'rhary*^ ^  -ay ^NoktoLKAUD Norwich hospa.tal
No. 382. Vol. XV. WITH EXTRA NURSING SUPPLEMENT. Saturday,
January 20th, 1894.

				

